# Evidence of Altered Peripheral Nerve Function in a Rodent Model of Diet-Induced Prediabetes

**DOI:** 10.3390/biomedicines8090313

**Published:** 2020-08-28

**Authors:** Md Jakir Hossain, Michael D. Kendig, Brandon M. Wild, Tushar Issar, Arun V. Krishnan, Margaret J. Morris, Ria Arnold

**Affiliations:** 1 School of Medical Sciences, UNSW Sydney, Sydney, NSW 2052, Australia; jakir.hossain@unsw.edu.au (M.J.H.); m.kendig@unsw.edu.au (M.D.K.); b.wild@unsw.edu.au (B.M.W.); m.morris@unsw.edu.au (M.J.M.); 2 Prince of Wales Clinical School, UNSW Sydney, Sydney, NSW 2052, Australia; t.issar@unsw.edu.au (T.I.); arun.krishnan@unsw.edu.au (A.V.K.)

**Keywords:** diabetic complications, peripheral neuropathy, prediabetes, nerve excitability, dyslipidemia, obesity, insulin resistance

## Abstract

Peripheral neuropathy (PN) is a debilitating complication of diabetes that affects >50% of patients. Recent evidence suggests that obesity and metabolic disease, which often precede diabetes diagnosis, may influence PN onset and severity. We examined this in a translationally relevant model of prediabetes induced by a cafeteria (CAF) diet in Sprague–Dawley rats (*n* = 15 CAF versus *n* = 15 control). Neuropathy phenotyping included nerve conduction, tactile sensitivity, intraepidermal nerve fiber density (IENFD) and nerve excitability testing, an in vivo measure of ion channel function and membrane potential. Metabolic phenotyping included body composition, blood glucose and lipids, plasma hormones and inflammatory cytokines. After 13 weeks diet, CAF-fed rats demonstrated prediabetes with significantly elevated fasting blood glucose, insulin and impaired glucose tolerance as well as obesity and dyslipidemia. Nerve conduction, tactile sensitivity and IENFD did not differ; however, superexcitability was significantly increased in CAF-fed rats. Mathematical modeling demonstrated this was consistent with a reduction in sodium–potassium pump current. Moreover, superexcitability correlated positively with insulin resistance and adiposity, and negatively with fasting high-density lipoprotein cholesterol. In conclusion, prediabetic rats over-consuming processed, palatable foods demonstrated altered nerve function that preceded overt PN. This work provides a relevant model for pathophysiological investigation of diabetic complications.

## 1. Introduction

The International Diabetes Federation (IDF) estimates that 451 million adults are currently living with diabetes while another 374 million are at high risk of developing diabetes [1]. Peripheral neuropathy (PN) is the most common microvascular complication of diabetes, with a lifetime prevalence exceeding 50% [2]. In diabetes PN causes pain, sensory loss and is a leading cause of ulceration and non-traumatic amputation, though a disease modifying treatment remains elusive [3]. A recent nationwide study in Denmark found that polyneuropathy in early onset type 2 diabetes (T2D) patients was associated with central obesity, hypertriglyceridemia, decreased high density lipoprotein (HDL) cholesterol, insulin resistance and other modifiable lifestyle factors [4]. Although PN is most prevalent in patients with established diabetes, evidence from both human and rodent studies suggests that the pathogenesis of PN is multifactorial and involves hyperglycemia as well as other metabolic derangements [5,6,7].

Given the paucity of effective treatments for PN [8], preclinical studies are critical for understanding its pathophysiology and developing successful therapies [8]. According to the ‘Neurodiab’ consensus guidelines, preclinical models of PN should be tailored to the diabetes type in question, while targeting appropriate neurological endpoints [9].

A key feature of ‘western’-style diets that foster over consumption and metabolic derangement is processed foods high in fat and refined sugar [10,11]. Therefore, we used a cafeteria-style diet (CAF), rich in fat and sugar [12] known to induce significant metabolic impairments in rats [13,14] to assess a number of PN endpoints.

Neuropathy phenotyping followed the Neurodiab guidelines by including all three key endpoints: electrophysiology, sensory behavior and anatomical markers of peripheral nerve damage [9]. In addition to standard electrophysiological measures, we utilized nerve excitability studies, which enable the indirect measurement of axonal ion-channel function and membrane potential [15]. Nerve excitability measures have proven sensitive to alterations in nerve function due to metabolic and toxic exposures [16,17] although they have not been extensively utilized in animal models. These measures demonstrate changes prior to nerve conduction abnormalities and thus represent a marker of early pathology [18,19].

## 2. Experimental Section

### 2.1. Animals and Diets

Thirty 7-week old male Sprague-Dawley rats (Animal Resource Centre, Perth, WA, Australia) were group-housed (3/cage) at 18–22 °C with a 12:12 h light: dark cycle (lights on 0700 h). Rats were acclimatized for one week and maintained (3 rats/cage) on ad libitum standard rat chow (Gordon’s chow, 11 kJ/g, 65% energy as carbohydrate, 22% protein and 13% fat) and potable water prior to dietary interventions. At 8 weeks of age rats were divided into weight-matched chow-fed control and CAF-fed prediabetic groups (chow 241.2 ± 3.2 (SEM); CAF 241.5 ± 3.9 g; *n* = 15/group). One rat from each group was excluded from final analyses as several of their adiposity measures were greater than 2 standard deviations above the group mean including: body weight, fat mass, plasma leptin, liver weight and blood triglycerides. All rats received standard rat chow and potable water; for CAF-fed rats this was supplemented with ad libitum access to a selection of cakes, biscuits, meat pies, dim sims and potato chips. CAF diet was varied daily, with the diet refreshed every afternoon. A minimum of two sweet and two savory items were provided daily [12]. The rats were maintained on their respective diets for 15 weeks. Animal procedures were approved by the UNSW Animal Care and Ethics Committee (Approval 17/111B, 1 September 2017) and were conducted in accordance with the Australian code for the care and use of animals for scientific purposes 8th edition.

### 2.2. Energy Intake, Body Weight and Body Composition Analysis

Food and water intake were measured twice weekly over 24 h commencing 3–5 p.m. by weighing individual foods and water. Twenty-four hours later foods and water were reweighed; intake was calculated from the weight difference, converted to kJ using manufacturers’ information and expressed as kJ/rat/24 h. Food intake analyses used cage as the unit of analysis (i.e., *n* = 5/group). Energy intake is reported per rat (assuming equal consumption by each rat in the cage). Body weight was measured twice per week. In week 13, body mass composition analysis was carried out with magnetic resonance imaging (Echo-MRI^TM^-900, Biological Resources Imaging Laboratory, UNSW Sydney, Sydney, Australia). Rats were placed in a plastic tube allowing minimal movement and scanned (2 min/rat).

### 2.3. Fasting Glucose and Intraperitoneal Glucose Tolerance Test (ipGTT)

Within a week of Echo-MRI, an intraperitoneal glucose tolerance test (ipGTT) was performed. Following an overnight fast, fasting blood glucose (FBG) was measured from the tail tip (Accu-Check^®^ Performa II blood glucose meter Roche, Mannheim, Germany). Rats were then administered 2 g glucose/kg lean mass (50% glucose solution in 0.9% NaCl), with blood glucose measured after 15, 30, 45, 60, 90, 120 and 180 min. The area under the curve (AUC) was expressed as mmol/L × min.

### 2.4. Lipids, Insulin, HOMA-Insulin Resistance, Leptin and Cytokines

Fasting triglycerides and HDL cholesterol were measured prior to ipGTT in whole blood from the tail tip with commercially available lipid panel test strips (PTS Diagnostics, Whitestown, IN, USA) and CardioChek^®^ PA analyser (PTS Diagnostics, Whitestown, IN, USA). A triglyceride glucose (TyG) index was calculated as ln ((fasting triglycerides (mg/dL) × fasting glucose (mg/dL)/2). Plasma collected at cull was used to measure fasting insulin and leptin using commercial enzyme-linked immunosorbent assay (ELISA) kits (Crystal Chem, Grove Village, IL, USA), and the inflammatory cytokines internleukin-6 (IL-6) and tumor necrosis factor-α (TNF-α; ABTS ELISA, Peprotech, Rocky Hill, NJ, USA). The homeostatic model assessment of insulin resistance (HOMA-IR) was calculated from terminal fasting insulin and FBG using the following formula: fasting insulin (milliunits/L) × FBG (mmol/L)/22.5.

### 2.5. Tactile Sensitivity

Tactile sensitivity was assessed by a blinded experimenter after 12 weeks of diet by stimulating the mid-plantar area of left and right hind paws with manual von Frey monofilaments (Stoelting, Wood Dale, IL, USA) using the up–down method [20,21]. Each filament was applied until paw withdrawal, with a cut-off duration of 8 s. Ten filaments ranging from 0.4 to 15 g were applied starting with the 2 g filament. At least 5 min separated the presentation of two filaments for each rat. If there was a positive response after the first filament presentation, the next lighter filament was applied while a negative response prompted the use of the next heavier filament. This up–down paradigm was continued until the responses of four more stimuli after the first change in response was obtained or the upper/lower end of the von Frey hair set was reached. When continuous positive or negative responses were observed until the weakest/strongest filament, values of 0.4 g (continuous positive responses) or 15 g (continuous negative responses) were assigned. The resulting pattern of positive and negative responses was tabulated and the 50% paw withdrawal threshold (PWT) was interpolated using the formula as described previously [20]. The test was repeated twice at 30 min intervals in both left and right paws and the average 50% PWT for both paws was analyzed.

### 2.6. Nerve Conduction and Nerve Excitability Studies

Electrophysiological assessment of peripheral nerve function was conducted on all 30 rats after 12 weeks of diet. Stimuli were delivered by an isolated stimulator (Model 2200 Analog Stimulus Isolator; A-M systems; Carlsborg, Washington, DC, USA), the signal was amplified and filtered (D440-2 isolated amplifier, Digitimer Ltd.; Welwyn Garden City, UK) and digitized with a data acquisition system (USB-6251; National instruments; Austin, TX, USA). Stimulating and recording electrodes were platinum needle electrodes (Natus; San Carlos, CA, USA).

#### 2.6.1. Nerve Conduction Studies (NCS)

NCS were undertaken in tail sensory nerve and sciatic motor nerve to measure nerve conduction velocity (NCV), amplitude and latency. Compound sensory nerve action potentials (SNAP) were obtained antidromically from tail sensory (caudal) nerves with the cathode positioned at the base of the tail, the anode at the thigh and recording electrodes placed in the skin of the tail 150 mm distal to the stimulating electrodes. Compound motor action potentials (CMAP) were obtained orthodromically from the sciatic nerve with the cathode positioned at the ankle, the anode at the base of the tail, with active and reference recording electrodes in the plantar muscles of the foot and in the plantar aspect of the 3rd digit respectively [22].

#### 2.6.2. Nerve Excitability

Nerve excitability was measured in the tibial motor nerve using the threshold tracking technique [22] in the hindlimb of anesthetized rats (induction 4% isoflurane and 1 L/min O_2_ flow rate then maintained under 2.5% isoflurane, approximately 30 min/rat). Tibial nerve stimulus was delivered with custom-made platinum needle electrodes with the cathode at the ankle and anode at the base of the tail. Compound muscle action potentials were recorded subcutaneously using needle electrodes from the plantar muscles of the foot.

Nerve excitability parameters were recorded using TRONDNF protocol with QTRACS software (^©^Institute of Neurology UCL, 2017, London, UK). This protocol included five testing paradigms yielding outputs of stimulus response behavior, strength duration relationship, threshold electrotonus (TE), current–threshold (I/V) and the recovery cycle [22,23]. Briefly, a maximal response is elicited using 1 ms stimuli that is incrementally increased; the software then establishes the stimulus required to generate a target response or ‘threshold’ (40% of maximal compound action potential). Deviations in the threshold were then measured following a range of conditioning stimuli including depolarizing and hyperpolarizing currents of up to 100 ms duration (TE) and up to 200 ms duration (I/V) [23,24]. The recovery cycle used a supramaximal conditioning stimulus with the threshold measured at multiple timepoints over the subsequent 200 ms. This parameter assesses the restoration of axonal excitability following a supramaximal stimulus and provides a measure of refractoriness (measured at 2.5 ms), superexcitability (measured at 5 ms) and subexcitability [23].

#### 2.6.3. Mathematical Modeling of Nerve Excitability Data

To identify drivers of change in nerve excitability, data was analyzed using the Bostock model of axonal excitability [25], as extended by Howells et al. [26]. This is a validated model of the human axon based on a single node and internode connected by pathways through and underneath the myelin sheath and has recently been applied in rodents [27]. The model identifies changes in maximal conductances or permeabilities of different types of sodium and potassium ion channels, alterations in sodium–potassium pump currents, or other biophysical properties. The model was first fitted to the mean nerve excitability in the control group before fitting the mean of the CAF group in a hypothesis-based approach. Modeling analysis involved changes in single or multiple membrane parameters in an iterative manner to objectively fit simulated nerve excitability data to the mean recorded data as closely as possible using a least squares approach. The ‘discrepancy’ between the simulated and recorded data was obtained by comparing the error between simulated and recorded data of the four excitability paradigms: strength–duration behavior, threshold electrotonus, current–threshold relationship and recovery cycle. Weighting factors of these paradigms were 0.5, 1, 1 and 3, respectively. Minimum interstimulus interval for the recovery cycle was set at 2.5 ms and analyses were run in unclamped mode to permit secondary changes in resting membrane potential caused by changes in conductances or pump currents.

### 2.7. Cull and Tissue Collection

After 15 weeks of diet, rats were fasted overnight, and after measuring FBG were deeply anaesthetized with ketamine (100 mg/kg) and xylazine (15 mg/kg) cocktail i.p. Naso-anal length and girth (at the xiphoid process) were measured and cardiac blood was collected into EDTA-coated tubes. After decapitation, the whole liver and the left retroperitoneal fat pad were weighed. The color and texture of the liver was scored on an ordinal scale ranging from 0 to 3, as previously reported [28]. Cardiac blood was centrifuged, and plasma stored at −30 °C. Length of the left tibia (cm) was measured. The Lee index (weight (g)^0.33^/naso-anal length (cm)) was calculated as a measure of adiposity. The right foot pad paw was dissected and fixed overnight at 4 °C in Zamboni fixative (2% paraformaldehyde and 0.2% picric acid in 0.1 M phosphate buffer). The tissue was washed in phosphate buffered saline (PBS) and placed in 20% sucrose with 0.1% Na-azide in PBS for 24 h then transferred to 30% sucrose solution in PBS and left at 4 °C.

### 2.8. Foot Pad Intraepidermal Nerve Fiber Density (IENFD) Analysis

The foot pad was embedded in OCT medium and 20 μm cryostat sections were cut. Five non-consecutive sections from the middle of the foot pad (*n* = 8/group) were stained. The sections were placed in a blocking solution for 1 h at room temperature (RT), then incubated overnight at 4 °C with rabbit polyclonal anti-human PGP9.5 antibody (ThermoFisher, Rockford, IL, USA, Catalog number #PA5-29012) diluted 1:500 in normal goat serum. The next day, sections were incubated in biotinylated goat anti-rabbit IgG (H+L) secondary antibody (Vector Lab, Burlingame, CA, USA, Catalog number #VE-BA1000) diluted 1:1000 in normal goat serum for an hour at RT. This was followed by quenching endogenous peroxidase activity for 30 min at RT followed by placing sections in extravidin peroxidase solution diluted 1:1000 in PBS for 30 min. Sections were placed in 3,3′-diaminobenzidine solution for 6–8 min for color development and then transferred to slides, air-dried, rinsed in water, dehydrated in 95% and 100% ethanol and cleared in xylene before mounting. Bright-field images were acquired with 20× objective. A minimum of 3–4 sections per rat were imaged with 3–6 fields acquired per section. The linear density (IENFD/mm) was quantified in Fiji-Image J following European Federation of Neurological Sciences guidelines [29]. All IENFD were quantified by a blinded experimenter.

### 2.9. Statistical Analyses

Results are presented as mean ± SEM. Group means were compared using independent samples *t*-tests when data passed the Shapiro–Wilk normality test; non-normal data were analyzed with non-parametric Mann–Whitney U test (GraphPad Software, version 7.04, San Diego, CA, USA). Body weight, energy intake and ipGTT data were analyzed by a two-way repeated measures ANOVA in IBM SPSS Statistics Software (Version 24, Armonk, NY, USA). Associations with nerve parameters and metabolic measures were determined by Pearson or Spearman correlations. Values of *p* < 0.05 were considered statistically significant.

## 3. Results

### 3.1. Metabolic Phenotype

**Figure 1:**
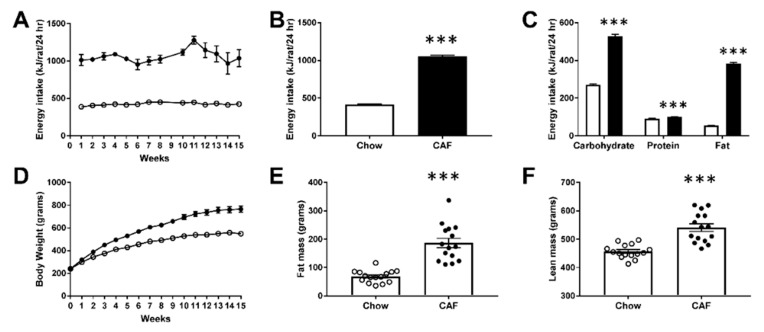
Effect of cafeteria (CAF) diet on energy consumption and body composition in rats. (**A**) Weekly energy intake (kJ/rat/24 h; two-way repeated measures ANOVA, F (1, 4) = 281.7, group (*p* < 0.001), time (*p* < 0.001) and interaction (*p* = 0.059), (**B**) average daily energy consumption in chow control and CAF rats shown as (kJ/rat/24 h; average of 27 measures) (unpaired t-test; *p* < 0.001), (**C**) breakdown of energy consumed from each macronutrient (kJ/rat/24 h; average of 27 measures; carbohydrate, protein and fat; all *p* < 0.001, Mann–Whitney test), (**D**) weekly body weights analyzed by two-way repeated measures ANOVA (group × time) showing main effect of group (F (1, 26) = 45.96, *p* < 0.001), time (F (15, 390) = 770.87, *p* < 0.001) and time × group interaction (F (15, 390) = 51.38, *p* < 0.001), (**E**) fat mass (grams) and (**F**) absolute lean mass (grams) in chow and CAF rats measured at 13 weeks of diet. The open circle or bar indicates chow group while the filled circle or bar indicates CAF group (*** *p* < 0.001 versus chow). *n* = 14/group in panels (**A**–**D**) and *n* = 15/group in panels (**E**,**F**).

CAF feeding significantly increased body weight and adiposity. Energy consumption in CAF-fed rats was significantly higher (2.5 fold) than chow rats over the study (*p* < 0.001, Figure 1A,B), with higher intake of carbohydrate, fat and protein (Figure 1C; all *p* < 0.001). CAF diet significantly increased fat mass and absolute lean mass at week 13 (*p* < 0.001; Figure 1E,F). At the end of the study, CAF rats were 39.5% heavier than controls (Table 1, *p* < 0.001) and exhibited significantly increased adiposity, growth, liver weight, liver score (indicating hepatic fat deposition) and fasting plasma leptin (Table 1). In keeping with previous work in HFD-fed rodents, there was evidence of dyslipidemia with significantly higher fasting blood triglycerides, TyG index and lower HDL cholesterol levels in CAF-fed rats (Table 1). CAF rats also exhibited increased plasma TNF-α while there were no significant changes in IL-6 (Table 1).

**Figure 2:**
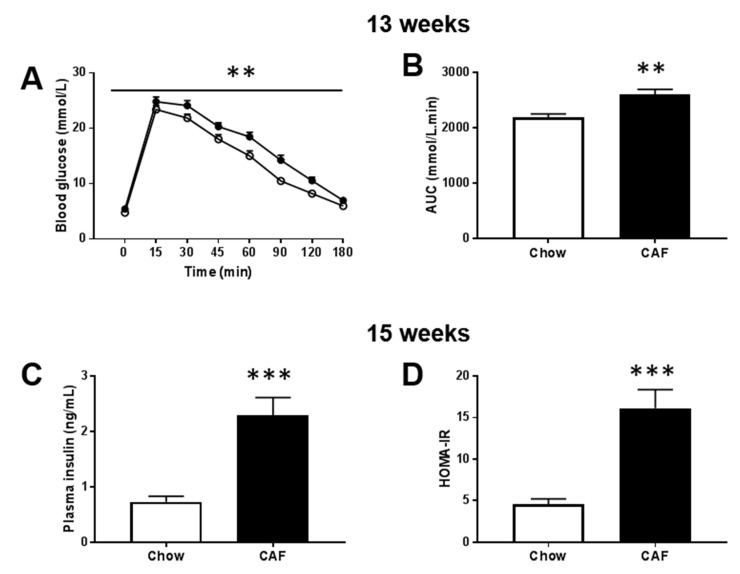
Intraperitoneal glucose tolerance test (ipGTT) at 13 weeks and plasma insulin concentrations and homeostatic model assessment of insulin resistance (HOMA-IR) at cull (15 weeks). (**A**) Glucose excursion curves up to 180 min of chow and CAF rats during ipGTT (*n* = 14 chow, 12 CAF; two-way ANOVA (group × time) indicated main effects of time (F (7, 168) = 365.32, *p* < 0.001) and group (F (1, 24) = 12.93, *p* = 0.001) and interaction (F (7, 168) = 2.061, *p* = 0.050). (**B**) Area under curve (AUC) for ipGTT (180 min; *n* = 14 chow, 12 CAF, *p* = 0.01; unpaired *t* test). Glucose injections were unsuccessful for two rats in the CAF group and thus did not generate data for a glucose response, leaving 14 chow and 12 CAF rats in panels A–B. (**C**) Plasma insulin concentrations (*n* = 14/group, *p* < 0.001). (**D**) HOMA-IR, marker of insulin resistance measured based on fasting glucose and insulin at cull (*n* = 14/group, *p* < 0.001). Insulin and HOMA-IR data were analyzed by a non-parametric Mann–Whitney U test. The open circle or bar indicates chow group while the filled circle or bar indicates the CAF group. (** *p* < 0.01; *** *p* < 0.001 versus chow).

Importantly for this study, the CAF group demonstrated glucose and insulin derangements consistent with prediabetes. Namely, elevated FBG, impaired glucose tolerance test (GTT) and high fasting insulin compared to controls. The GTT performed at 13 weeks of diet revealed higher FBG in CAF-fed rats compared to chow-fed control rats (5.4 ± 0.1 vs. 4.8 ± 0.1; *p* < 0.001), impaired glucose clearance (Figure 2A) and greater AUC over180 min (Figure 2B; *p* < 0.01). At study endpoint, FBG remained significantly higher in the CAF group (Table 1), as were fasting insulin and HOMA-IR index (Figure 2C,D; *p* < 0.001).

### 3.2. Peripheral Nerve Function and Neuropathy Phenotype

**Figure 3:**
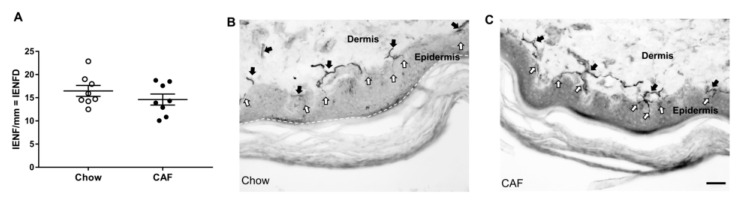
Foot pad intra-epidermal nerve fiber density (IENFD) in rats. (**A**) Mean IENFD in plantar footpad sections in chow and CAF rats stained with PGP9.5. (minimum of 3–4 sections/rat and 3–6 fields/section were counted; *n* = 8/group, unpaired t-test, *p* = 0.285). (**B**,**C**) Representative images from chow and CAF rat plantar footpad sections stained with PGP9.5. The white dotted line shows the border of the epidermis. The thick dark structures (black arrow) lying horizontally are the nerve bundles and the thin lines (white arrow) originating from the nerve bundles and vertically extending towards the epidermis were counted as intraepidermal nerve fibers (Image—20×, scale bar 50 μm).

CAF-fed and control rats did not differ on conventional nerve conduction measures including amplitude, conduction velocity and latency, for sensory or motor nerves (all *p* > 0.05, Table 2). Nerve excitability measures taken from the tibial motor nerve, however, demonstrated a significant increase in superexcitability at the 5 ms interstimulus interval (*p* < 0.05, Table 2, Figure 3A). There were no group differences on other measures of nerve excitability—strength–duration time constant, threshold electrotonus, resting I/V slope, refractoriness and subexcitability (Table 2).

**Figure 4:**
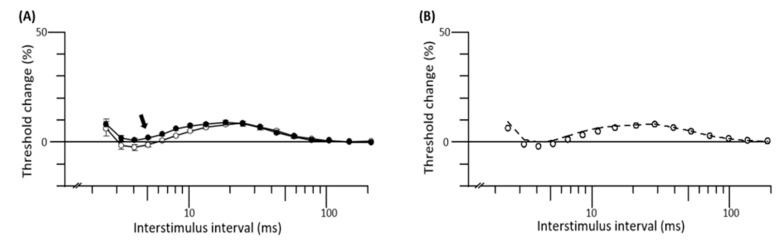
Tibial nerve recovery cycle graphs of mean group data and mathematical modeling. (**A**) Graph of the mean group recovery cycle data (open circles represent chow group and closed circles represent CAF group). Superexcitability was measured at 5 ms indicated by the arrow. (**B**) Mathematical modeling of nerve excitability reduced the discrepancy between groups with decreased sodium–potassium pump current.

Tactile sensitivity assessed using von-Frey monofilaments after 12 weeks of diet showed no difference in 50% paw withdrawal threshold in CAF compared to chow rats (Table 2). Similarly, quantification of IENFD in foot pad sections after 15 weeks of diet revealed no difference between CAF and chow rats (Figure 4A–C).

### 3.3. Mathematical Modelling of Nerve Excitability

Mathematical modeling indicated that the nerve excitability differences between groups were best explained by a reduction in sodium–potassium pump current (CAF −15: Chow 0 picoamperes; Figure 3B). The single change in this parameter alone reduced the discrepancy between groups by 66%, suggesting altered sodium–potassium pump current explained most of the group difference. The model was not substantially improved by adding changes in any other single parameter (all <10% improvement).

### 3.4. Correlations between Nerve Parameters and Metabolic Measures

**Figure 5:**
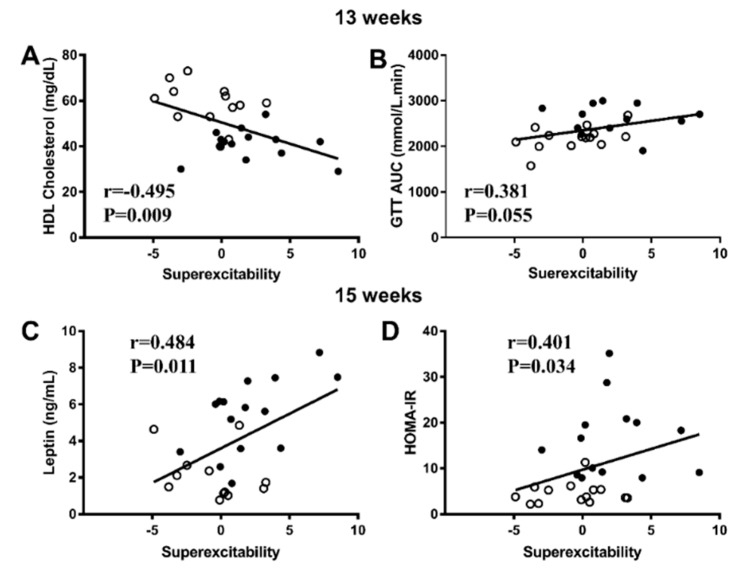
Correlations between superexcitability and metabolic parameters at 13 weeks and 15 weeks of diet. (**A**,**B**) Correlations between superexcitability and fasting blood HDL cholesterol and glucose tolerance test (GTT) AUC 180 min at 13 weeks of diet and (**C**,**D**) correlations between superexcitability and fasting plasma leptin and HOMA-IR calculated based on fasting blood glucose and fasting plasma insulin concentrations at 15 weeks of diet. Data are presented as scatterplots of individual values where open points indicate chow rats and black points indicate CAF rats. HDL, GTT and leptin data were analyzed by Pearson correlations whereas HOMA-IR was analyzed by Spearman correlation, *n* = 26–28.

Correlational analyses (Figure 5) indicated that superexcitability correlated significantly with measures of adiposity assessed by EchoMRI within 1 week of nerve testing (13 weeks). Specifically, superexcitability correlated positively with fat mass (*r* = 0.497; *p* = 0.007), body weight (*r* = 0.483; *p* = 0.009) and lean mass (*r* = 0.469; *p* = 0.012). These three variables were closely interrelated (all *r* > 0.88; *p* < 0.001) and should be considered together. Increased superexcitability also significantly correlated with lower HDL (*r* = −0.495; *p* = 0.009, Figure 5A) but not triglycerides (*r* = 0.154, *p* = 0.453). Fasting blood glucose was not associated with superexcitability, though a trend for GTT AUC at 180 min was noted (*r* = 0.381; *p* = 0.055, Figure 5B). Tail sensory nerve amplitude (SNAP) also correlated negatively with FBG measured at 13 weeks (*r* = −0.454, *p* = 0.015, *n* = 28).

In keeping with the correlations observed at 13 weeks, positive correlations between superexcitability and measures of weight, adiposity and HDL persisted at cull including rpWAT mass (0.506, *p* = 0.004), fasting plasma leptin (*r* = 0.484, *p* = 0.011, Figure 5C), HOMA-IR (Spearman *r* = 0.401, *p* = 0.034, Figure 5D) and fasting plasma insulin (Spearman *r* = 0.379, *p* = 0.047), but not inflammatory markers TNF-α (*r* = 0.373, *p* = 0.088) or IL-6 (*r* = 0.200, *p* = 0.349). Finally, there were significant negative correlations between IENFD and fasting plasma triglycerides (*r* = −0.527, *p* = 0.036, *n* = 16) and the TyG index (*r* = −0.527, *p* = 0.043, *n* = 16).

## 4. Discussion

This study demonstrated altered nerve excitability in CAF-fed prediabetic rats compared to chow-fed controls. The nerve excitability abnormalities occurred in the absence of changes in nerve conduction values and IENFD, suggesting that axonal dysfunction in this model is apparent before structural abnormalities appear. Mathematical modeling of nerve excitability data suggested that axonal dysfunction was related to reduced sodium–potassium pump current. The study also demonstrated that these neurophysiological changes were correlated with measures of adiposity, HDL cholesterol and insulin resistance, suggesting that metabolic changes may be drivers of early axonal dysfunction.

HFD feeding (45–60% kJ as fat) is commonly utilized to model human prediabetes or diet-induced obesity in rodents, and recapitulates several aspects of PN [30,31,32]. However, no previous studies have characterized PN using a model that induces prediabetes using a palatable CAF diet rich in fat and simple sugars commonly consumed as part of the western diet. CAF rats met criteria for prediabetes with elevated FBG, insulin resistance and glucose intolerance as well as several criteria for metabolic syndrome including, increased adiposity, hypertriglyceridemia and reduced HDL cholesterol. These findings replicate the complex range of risk factors demonstrated to increase PN risk in humans [33], highlighting the translational relevance of the model. While at 13 weeks of diet there was no change in nerve conduction, there was a significant increase in the nerve excitability parameter, superexcitability, in the CAF-fed group. Nerve excitability parameters are an electrophysiological measure of axonal membrane and ion channels and thus this finding indicates early functional change in the peripheral axon [24].

Mathematical modeling of nerve excitability data suggested that the difference in superexcitability was mediated by a reduction in sodium–potassium pump current. Sodium–potassium pump function in neurons and Schwann cells is highly dependent on the availability of cellular ATP and stability of energy supply [34]. A hallmark of obesity and metabolic syndrome is lipid and glucose dysregulation; these extrinsic forces result in substantial intrinsic cellular dysfunction in peripheral nerves via a cascade of events that impair energetic status through mitochondrial damage, oxidative and endoplasmic reticulum stress [35]. Taken together, these changes may explain the difference in superexcitability between CAF and chow rats. Future studies undertaking experimentation and quantitation of peripheral nerve sodium potassium pump activity are necessary to substantiate this hypothesis.

The changes in axonal function demonstrated in this model were evident without accompanying deficits in nerve conduction values. This finding is in keeping with several clinical studies that have demonstrated that changes in motor nerve excitability occur prior to overt PN [18,36,37,38]. Increased sensory nerve superexcitability has been demonstrated in human prediabetes, which correlated with glycated hemoglobin in the absence of changes in other nerve excitability parameters and NCV measures suggesting that such isolated findings in one parameter has physiological meaning [39]. We did not study sensory nerve excitability since sensory electrophysiological studies in rats are typically limited to the tail and sensory nerve excitability of the rat tail is distorted by low level compound muscle action potential activation that occurs due to conditioning stimuli in the nerve excitability protocol [40]. Despite this, changes in motor nerve excitability were evident in our cafeteria-fed rats, supporting its utility as an early marker of pathological change in metabolic syndrome and prediabetes.

Critically, increased superexcitability was associated with a poorer metabolic profile in terms of body weight, fat mass and fasting plasma leptin. The relationships between nerve dysfunction and measures of adiposity in our study add to mounting clinical evidence that obesity is a driving factor in the development of PN [33,41,42,43]. Moreover, blood HDL measured at 13 weeks was negatively related to axonal dysfunction. A study in humans found that reduced HDL cholesterol was the strongest inverse metabolic correlate in metabolic syndrome neuropathy and was correlated negatively with sensory symptoms, signs and sural nerve amplitudes [44]. Experimental evidence in HFD fed mice [5,32,45,46] and rats [47,48,49] also revealed that obesity and dyslipidemia are major players in inducing PN. However, there are some inconsistencies regarding the severity of PN in different diet-induced obesity models which may result from the source and type of fat content of the HFD, the duration of feeding, differences in sex and age of the animals and different approaches employed for PN phenotyping [31]. More studies are needed to elucidate the underlying mechanisms of HFD induced nerve dysfunction. One study showed that oxidized low-density lipoprotein (oxLDL) induced oxidative stress was elevated in the sciatic nerve of HFD fed mice, which subsequently developed NCV and sensory deficits [45]. They also found that in vitro, oxLDLs increased oxidative stress in dorsal root ganglion (DRG) sensory neurons [45]. Another study reported increased sorbitol pathway activity with accumulation of glucose, sorbitol and fructose in the sciatic nerve due to HFD feeding in mice and demonstrated elevated oxidative-nitrosative stress in the sciatic nerve and DRG neurons [32]. The influence of specific dietary components such as saturated fatty acids have also been implicated in murine models of PN with demonstrable alterations in DRG mitochondrial function, ATP production and apoptosis [50,51]. Notably, the diet used here is rich in saturated fat, making these mechanisms highly relevant and worthy of further investigation. Our work reinforces the importance of HFD induced obesity or dyslipidemia in triggering PN and, for the first time, demonstrates a relationship between nerve excitability and components of metabolic syndrome prior to NCV and sensory deficits in a rat model.

While HDL parameters correlated most strongly with superexcitability, there was a trend toward a relationship with glucose intolerance (assessed using AUC), suggesting a subtle impact of glucose dysregulation as well. This was supported by significant relationships between insulin resistance and superexcitability. Previous studies in HFD induced obese mice [5,32,45] and rats [52,53,54] demonstrated that two key components of the metabolic syndrome, insulin resistance and glucose intolerance could contribute to PN. Insulin is a neurotrophic factor and loss of this neurotrophic support due to insulin resistance as seen in metabolic syndrome and T2D, is thought to affect PI3K/Akt signaling pathway inside the neurons leading to mitochondrial dysfunction and oxidative stress, thus promoting PN [55]. In metabolic syndrome and prediabetes, continued excess energy intake (as seen here) creates a vicious feed-forward cycle where insulin resistance initiates chronic inflammation, which in turn exacerbates insulin resistance and promotes an injury cascade within the neuron and nerve environment [35]. Here we observed an elevation in plasma inflammatory markers in CAF rats, though this did not correlate with neuropathy measures. Molecular analysis of the sciatic/tibial nerve in future studies may prove informative.

In this study, no difference was observed between groups in tactile sensitivity, contrasting previous studies in rodents that have reported decreased mechanical paw withdrawal threshold following HFD feeding [32,49,56,57]. However, in those studies, increased tactile sensitivity was accompanied by changes in NCV measures while in our rats NCVs were not affected. Similarly, IENFD was not different between groups in our study. We speculate that a longer duration of diet may be required to induce overt structural neurological deficits as previous studies in HFD fed rats found IENFD to be decreased after 24 weeks of diet [56,58] while other work in mice showed no change in IENFD after 12–16 weeks of HFD [32,59]. Despite no group differences, IENFD correlated negatively with plasma triglycerides and TyG index, supporting the notion that the aetiology of PN is closely related to dyslipidemia [7].

## 5. Conclusions

Our study is the first to examine peripheral nerve function in a model of metabolic syndrome and prediabetes induced by a palatable cafeteria-style diet. We observed altered peripheral nerve function using nerve excitability measures, which correlated with multiple metabolic parameters. Notably, these abnormalities occurred in the absence of changes in nerve conduction and structural nerve damage. The functional nerve changes demonstrated here suggest that nerve excitability could be a useful measure of early neuropathic changes in animal models of diabetic neuropathy. Future studies using a longer duration of diet or more severe diabetic phenotypes may assist in establishing whether these functional nerve changes precede the development of overt neuropathy.

## Figures and Tables

**Table 1:** Blood lipids (13 weeks) and endpoint measures (15 weeks).

Parameters	Chow	CAF	*p* Value
Blood Lipids (13 weeks)			
Triglycerides (mg/dL)	116.0 ± 7.3	157.0 ± 9.5	0.003
HDL Cholesterol (mg/dL)	58.23 ± 2.61	40.93 ± 1.80	<0.001
TyG Index	9.87 ± 0.08	10.30 ± 0.08	<0.001
Terminal Data (15 weeks)			
Body Weight (g)	548 ± 11	765 ± 26	<0.001
Naso-anal Length (cm)	26.7 ± 0.3	28.2 ± 0.2	<0.001
Circumference (cm)	19.9 ± 0.2	22.8 ± 0.6	<0.001
Lee Index	0.307 ± 0.002	0.324 ± 0.003	<0.001
Tibia Length (cm)	4.4 ± 0.03	4.5 ± 0.03	0.003
rpWAT (g)	4.0 ± 0.4	14.3 ± 1.2	<0.001
Liver Weight (g)	14.1 ± 0.4	18.9 ± 0.7	<0.001
Liver Score (0–3)	0.36 ± 0.13	2.50 ± 0.17	<0.001
FBG (mmol/L)	5.0 ± 0.1	5.5 ± 0.1	0.006
Fasting Plasma Leptin (ng/mL)	2.1 ± 0.4	5.7 ± 0.5	<0.001
Plasma IL-6 (pg/mL)	891.0 ± 52.2	1173.1 ± 143.4	0.084
Plasma TNF-α (pg/mL)	1418 ± 115	2761 ± 563	0.036

Notes: HDL—high-density lipoprotein, TyG—Triglyceride glucose, rpWAT—retroperitoneal white adipose tissue, FBG—fasting blood glucose, IL—Interleukin and TNF—Tumor Necrosis factor. Data were analyzed by an independent sample *t* test except for the Lee index, tibia length, liver score and plasma leptin, which were non-parametric and were analyzed by a Mann–Whitney test. *n* = 12–14 for all parameters, except IL-6 (chow = 11) and TNF-α (chow = 9).

**Table 2:** Manual von Frey results and parameters of peripheral nerve function obtained with electrophysiological assessments.

Parameters	Chow	CAF
Von-Frey 50% PWT (g)	2.99 ± 0.39	3.98 ± 0.62
Nerve Conduction Studies		
Tail SNAP (μV)	19.06 ± 1.73	19.91 ± 2.64
Tail SNCV (m/s)	34.01 ± 0.87	33.25 ± 0.84
Tibial Amplitude (mV)	7.49 ± 0.52	7.60 ± 0.64
Sciatic MNCV (m/s)	42.31 ± 1.59	40.43 ± 1.94
Nerve Excitability Indices		
TEd 10–20 ms (%)	37.48 ± 0.81	36.01 ± 0.95
Resting I/V (slope)	1.04 ± 0.03	1.06 ± 0.04
Refractoriness at 2.5 ms (%)	6.20 ± 1.63	7.81 ± 1.24
Superexcitability at 5 ms (%)	−0.65 ± 0.68	2.14 ± 0.83 *
Subexcitability (%)	8.16 ± 0.69	8.00 ± 0.96

* (*p* < 0.05 versus control); SNAP—Sensory nerve amplitude, SNCV—Sensory nerve conduction velocity, MNCV—Motor nerve conduction velocity, Ted—Threshold electrotonus to depolarizing current and I/V—Current/threshold. All data were analyzed by an independent sample *t* test except for the von Frey, which was analyzed by a Mann–Whitney test. *n* = 12–14 for all data.
